# Associations Between Patient Neighborhood Characteristics and Inappropriate Antimicrobial Use

**DOI:** 10.1017/ash.2021.73

**Published:** 2021-07-29

**Authors:** Joseph Engeda, Jane Kriengkauykiat, Erin Epson

## Abstract

**Background:** Antimicrobials are among the most commonly prescribed medications in US hospitals; an estimated 50% of hospitalized patients receive an antimicrobial. Research has shown that antimicrobial prescriptions to vary by patient- and hospital-level factors; however, disparities by patient neighborhood characteristics have not been examined. We evaluated associations between hospital and neighborhood indicators of socioeconomic status (SES) and antimicrobial use (AU) for gram-positive bacterial infections (GPBs), and broad-spectrum use for community-acquired infections (BSCAs) and hospital-onset infections (BSHOs). **Methods:** This analysis was conducted among 86 acute-care hospitals in California that submitted AU data via the NHSN in 2019. Hospital-level AU was measured as standardized antimicrobial administration ratios (SAARs) calculated by dividing observed antimicrobial use by risk-adjusted predicted antimicrobial use for GPB, BSCA, and BSHO antimicrobial groupings and categorized as binary (>1 or <1); SAARs >1 indicate potential inappropriate prescribing. California Office of Statewide Health Planning and Development 2018 data were used to obtain hospital characteristics and patient age, race or ethnicity, insurance, and comorbidities (defined by Charlson comorbidity index) for hospitalizations where AU may have been indicated, based on *International Classification of Diseases Tenth Revision* (ICD-10) diagnosis codes. The California Healthy Places Index (HPI) was used to obtain composite neighborhood SES indicators for each patient at the ZIP code level, measured as tertiles. Covariates were aggregated to the hospital level. Poisson regressions were used to evaluate the association between hospital and neighborhood SES indicators and SAAR scores, controlling for potential hospital-level confounders. **Results:** Among 86 hospitals included in the analysis, the mean patient age for hospitalizations where AU may have been indicated was 66 years, the proportion of white patients was 55%, and the mean proportion of Medi-Cal users was 19%. After adjusting for confounders including age, race or ethnicity, insurance status, comorbidities, and number of hospital beds; higher median values of patient SES had a protective effect against hospitals having GP SAAR scores > 1 (relative risk [RR], 0.68; 95% CI, 0.50–0.93) but was not significantly associated with hospitals having BSCA SAAR scores >1 (RR, 0.79; 95% CI, 0.62–1.02) or BSHO SAAR scores >1 (RR, 0.80; 95% CI, 0.61–1.04). **Conclusions:** Considering SES in addition to summary antimicrobial use scores such as SAARs may help identify populations potentially at risk for inappropriate AU; however, patient-level information is still necessary to evaluate appropriateness of antimicrobial prescribing.

**Funding:** No

**Disclosures:** None

